# Brachial-Ankle Pulse Wave Velocity Is the Only Index of Arterial Stiffness That Correlates with a Mitral Valve Indices of Diastolic Dysfunction, but No Index Correlates with Left Atrial Size

**DOI:** 10.1155/2013/986847

**Published:** 2013-03-06

**Authors:** Bryan Chow, Simon W. Rabkin

**Affiliations:** Department of Medicine (Cardiology), University of British Columbia, Level 9, 2775 Laurel Street, Vancouver, BC, Canada V5Z 1M9

## Abstract

The objective of this study was to determine the optimal assessment of arterial stiffness that relates to diastolic dysfunction. Forty-one patients had measurements of brachial-ankle pulse wave velocity (baPWV), carotid-femoral pulse wave velocity (cfPWV), ankle brachial index (ABI), pulse pressure (PP), and augmentation index (AIx). Diastolic dysfunction was evaluated by echocardiographic indices of the ratio of the peak early diastolic mitral valve velocity and the peak late diastolic velocity (*E/A* ratio), left atrial diameter, and left atrial volume indexes. There was a significant (*P* < 0.05) correlation between baPWV and *E/A* ratio with an inverse relationship indicating that higher arterial stiffness was associated with greater diastolic dysfunction. In contrast, there was no significant correlation between *E/A* ratio and cfPWV, PP, ABI, or AIx. After multivariate analysis, the relationship between baPWV and *E/A* ratio remained significant (*P* < 0.05), independent of age and systolic blood pressure (BP). There were no correlations between any index of vascular stiffness and left atrial dimension or volume. In summary, baPWV correlates with diastolic dysfunction, independent of a patient's age and BP and is a better indicator of diastolic dysfunction than other indicators of arterial stiffness. baPWV has the utility of infering the presence of left ventricular diastolic dysfunction.

## 1. Introduction

Compromised left ventricular diastolic function represents an ongoing cardiovascular challenge from the perspective of its detection, assessment, and management [1, 2]. The early detection of diastolic dysfunction has garnered increasing attention because it predicts the development of heart failure [3, 4]. Currently, the primary imaging modality for assessing left ventricular diastolic function is Doppler echocardiography although there are controversies in the precision of its assessment [5–8]. It can require the integration of transmitral flow, pulmonary venous flow, mitral annular motion, myocardial deformation, and cardiac structure. This complex measurement of diastolic function can be reduced to several common elements—the assessment of transmitral flow velocity and evaluation of cardiac structure which includes left atrial size [1, 8]. The ratios of peak early diastolic mitral flow filling velocity (*E*) and of the peak late mitral diastolic filling velocity (*A*), *E/A* ratio, characterize the severity and stage of the diastolic dysfunction [9]. In diastolic dysfunction, the *E/A* ratio decreases as *E* velocity is reduced and *A* velocity is increased [9]. Because ageing alters the *E/A* ratio, age adjustment is necessary [6]. Another component of the assessment of diastolic dysfunction is left atrial (LA) dimension. LA size or volume has been proposed as a simple noninvasive assessment of the degree of LV diastolic dysfunction [10]. LA size is increased, and LA emptying is decreased in patients with diastolic heart failure [11].

While the role of arterial stiffness in prediction of cardiovascular events is well documented [12–15], the capacity of arterial stiffness to alter left ventricular diastolic function is less certain. Arterial stiffness is hypothesized to produce LV diastolic dysfunction through increased cardiac after load [16, 17] and reduced coronary perfusion; the latter is hypothesized to produce LV diastolic dysfunction through decreased diastolic blood pressure [18]. The concept that systolic arterial loading can impair subsequent diastolic function, however, was not substantiated by investigators using the more direct assessment of myocardial relaxation by examining LV pressure decay during early diastole or tau [19]. In addition, the proposition that arterial stiffness impairs left ventricular relaxation and subsequent diastolic dysfunction has had mixed clinical evidence, with some studies supporting the presence of an association [20], some are equivocal [21], while other studies found no relationship [22]. Some of the studies that reported a relationship between arterial stiffness and diastolic dysfunction did not adjust for factors such as age [20] that affect arterial stiffness and diastolic dysfunction independently.

Variations in reports may in part be due to differences in techniques for the measurement of arterial stiffness. Pulse wave velocity (PWV) is a validated method to quantify arterial stiffness [23] and can be measured in different arterial segments, with brachial-ankle PWV (baPWV) and carotid-femoral PWV (cfPWV) being the most common ones. Augmentation index (AIx), pulse pressure (PP), and increased ankle brachial index (ABI) are also indices of arterial stiffness. AIx has traditionally been considered to represent increased wave reflection [24], while more recently increased AIx has been attributed to a reduction in the reservoir function—compliance properties of the aorta and other elastic arteries [25]. Increased arterial stiffness produces an increase in systolic blood pressure and reduction in diastolic blood pressure which is easily recognized by an increase in pulse pressure [26]. Increased ABI, occurring in the absence of peripheral arterial disease, indicates a greater augmentation in systolic arterial pressure as the pulse wave moves the longer distance to the ankle arteries compared to the shorter distance to the brachial arteries, and it is due to an increase in arterial stiffness [27]. Which of these indices of arterial stiffness, if any, best identifies a relationship with diastolic dysfunction needs to be determined?

The objective of this study was to determine whether arterial stiffness is related to left ventricular diastolic dysfunction and to determine which of four indices of arterial stiffness—baPWV, cfPWV, ABI, or pulse pressure—correlated best with an echocardiographic assessment of diastolic dysfunction.

## 2. Methods

Patients from a cardiology clinic at a University teaching hospital who met the following criteria were studied. Men or women over the age of 45 years that agreed to participate in the study were included. Patients were only included if they had a satisfactory echocardiographic assessment without significant aortic or mitral valve diseases or impairment in left systolic ventricular function identified on echocardiographic assessment.

A noninvasive measure of vascular stiffness was performed as previously outlined [27, 28]. PWV and augmentation index were measured on an Omron Colin VP1000/2000 (Ill, USA). Briefly, the subject was examined while resting in the supine position, with electrocardiogram electrodes placed on both wrists and a microphone for detecting heart sounds placed on the left edge of the sternum. Blood pressure cuffs, which were connected to the device, were placed around both arms and ankles. After the participants rested in the supine position, the right carotid pulse wave and femoral artery contours were recorded simultaneously by placement of a transducer over these arteries. Using the carotid pressure waveform, the augmentation index (AIx) was calculated as the difference between the second and the first systolic shoulder and was expressed as a percentage of the carotid pulse contour. PWV was calculated as the ratio of the distance in meters from the brachial artery to ankle or carotid to femoral artery and the transit time in seconds, measured from the basal level of one pulse to the other either baPWV or cfPWV.

Cardiac structure and function were assessed according to a standardized protocol. Two-dimensional echocardiograms were performed. Parasternal long- and short-axes views were used to determine left ventricular end-diastolic and end-systolic measurements and wall thickness dimensions and left atrial diameter according to the recommendations of the American Society of Echocardiography [29]. Diastolic function was determined using Doppler assessment of mitral inflow velocity specifically peak early and late diastolic mitral flow velocities. Left atrial volume was measured using the biplane area-length method determined from the apical 4 chamber view. Left atrial volume was indexed by body surface area. All measurements were recorded by an ultrasonographer unaware of the clinical condition of the participants. 

All numeric data are expressed as mean values (±SD), and categorical data are present at percentages. Univariate and multivariate regression analyses were conducted using PASW Statistics 18. Statistical significance was set at *P* < 0.05.

## 3. Results

**Figure 1:**
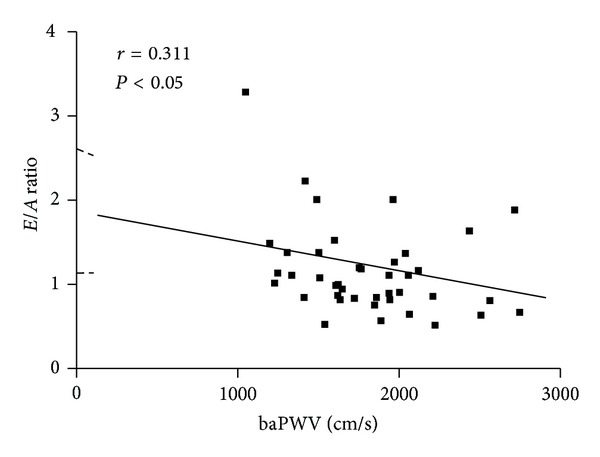
The relationship between baPWV and *E/A* ratio.

The study population consisted of 41 adult patients (mean age 68 ± 11 years, 66% male) of whom 73% had hypertension and 12% had diabetes mellitus (Table 1). The echocardiographic data indicate that the group had a normal systolic function with an ejection fraction of 63.3 ± 5%. There was a significant correlation between baPWV and *E/A* ratio (Figure 1). The relationship was negative, indicating a decreasing *E/A* ratio with increases in baPWV. None of the other indices of arterial stiffness, cfPWV, ABI, or Aix, correlated significantly with *E/A* ratio (Table 2). Measures of left atrial size, left atrial diameter (LAD) and left atrial volume index (LAVI), did not correlate significantly with any index of arterial stiffness. LAD correlated significantly with age. The number of subjects without hypertension is small and precluded meaningful separate analysis.

**Figure 2:**
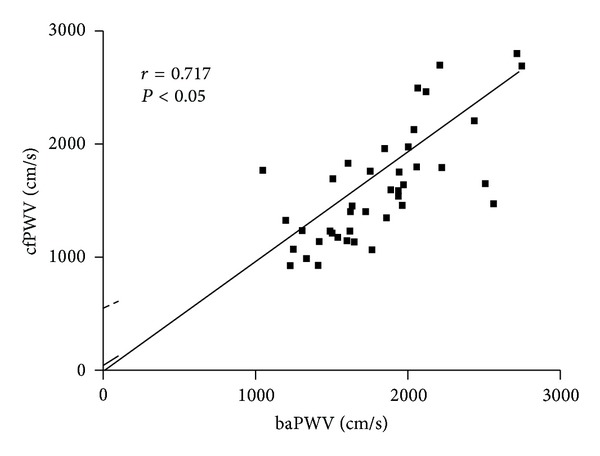
The relationship between baPWV and cfPWV.

Because baPWV and cfPWV comprise some of the same elements of arterial stiffness, the relationship between baPWV and cfPWV was explored by a linear correlation analysis. There was a significant relationship between the two factors with an *r* value of 0.717 or *r* ^2^ value of 0.514 (Figure 2). The line of identity demonstrates that baPWV exceeds cfPWV on a consistent basis. baPWV was 17% greater than cfPWV.

Age was positively correlated with baPWV (*P* < 0.001) and cfPWV (*P* < 0.001). There were also significant positive correlations between systolic blood pressure (SBP) and both baPWV (*P* = 0.044) and cfPWV (*P* = 0.002). Pulse pressure was significantly correlated with cfPWV (*P* = 0.007), but IT did not correlate significantly with baPWV or AIx. There was a significant positive correlation between age and pulse pressure (*P* = 0.015).

The relationship between age and indices of aortic stiffness necessitates the use of multivariate analysis in order to consider the potential effect of age on the relationship between aortic stiffness and *E/A* ratio. A significant association between baPWV and *E/A* ratio persisted after adjusting for age, SBP, and PP in a multiple regression analysis (Table 3). Multivariate analysis was utilized, in case one of the variables was obscuring a relationship between cfPWV and *E/A* ratio. Age and SBP were significantly correlated with cfPWV in multivariate analysis. There was no significant relationship between cfPWV and *E/A* ratio after multiple regression analysis (Table 4).

## 4. Discussion

The major contribution of this study is the comparative analysis of different indices of arterial stiffness and their association with left ventricular diastolic dysfunction. We found that baPWV was significantly correlated with an indicator of diastolic dysfunction, and the association was independent of age and blood pressure. Importantly, baPWV was a better predictor of diastolic dysfunction than several other indices of arterial stiffness, namely, cfPWV, AIx, PP, or ABI. 

Both baPWV and cfPWV are indicators of arterial stiffness, and each of them is related to cardiovascular morbidity and mortality [14, 15, 30–32]. With regards to diastolic dysfunction, recent investigations have mainly examined either baPWV [22, 33–37] or cfPWV [20, 38–41] in relation to *E/A* ratio. We compared baPWV and cfPWV and found a significant relationship between diastolic dysfunction and baPWV but not with cfPWV. It was not our purpose to compare baPWV versus cfPWV over the spectrum of cardiovascular diseases; we rather focused solely on the association with an index of left ventricular diastolic function and found a greater utility for baPWV. While both baPWV and cfPWV are indicators of arterial stiffness, baPWV is an indicator of the combination of central and peripheral arterial stiffness [31, 42–44]. Our study suggests that the effects of peripheral arterial stiffness on ventricular diastolic function are better represented by the greater arterial path measured by baPWV as compared to the centrally confined path of cfPWV. While baPWV and cfPWV are highly and significantly correlated with each other, the correlation is far from being identical. We found a *r* ^2^ of 0.513, indicating that only about 50% of the variance of one is accounted for by the other. This value is similar to the one reported by others (*r* ^2^ = 0.63) [42]. We found that baPWV was predominantly greater than cfPWV by 17%. This is consistent with another report that shows that, on average, baPWV was approximately 20% higher than cfPWV [42]. The higher value presumably reflects the additional contribution of “peripheral” (or muscular) arterial stiffness to central arterial stiffness [43]. baPWV recognizes a different vascular stiffness pathway, and, is one differently affected by interventions such as weight loss [45]. The association of baPWV with an increased risk of total cardiovascular events and all-cause mortality [15] may reflect the effect of baPWV on the heart.

Our finding that baPWV correlates with LV diastolic function is consistent with several studies [34, 35, 37] but they did not compare baPWV with a wide range of other measurements of arterial stiffness. Wang et al. compared PWV measurements in the brachial-ankle, carotid-femoral, heart-femoral, and heart-carotid segments among hypertensive patients and also found baPWV to be the best predictor of diastolic dysfunction amongst measurements of PWV [46]. However, they did not conduct a multivariate analysis adjusting for age and blood pressure [46]—two factors that influence diastolic cardiac function. 

Age and systolic blood pressure are the two primary determinants of baPWV and cfPWV as we found and as has been reported previously [42]. Therefore, our measurements of PWV were adjusted for these two parameters. With advancing age, arterial stiffness increases due to changes in arterial elasticity and compliance, leading to elevations in systolic pressures and pulse pressure [47–49]. Prolonged elevations in blood pressure can also independently lead to similar changes in the vasculature that increases arterial stiffness [50, 51]. The associations of age, SBP, and PP with baPWV and cfPWV are confirmed in the present study. After including these factors in multivariate analysis, the negative relationship between baPWV and *E/A* ratio remained significant, and the baPWV relationship with mitral valve velocity measurements was independent of age, SBP, and PP.

We did not find any associations between indices of arterial stiffness and left atrial size assessment either by left atrial diameter or left atrial volume. While left atrial size has been purported to be a marker of chronic diastolic dysfunction and cardiovascular disease risk [52], previous data on the relationship of left atrial size and arterial stiffness have been limited and somewhat conflicting. One study reported no significant association between PWV or pulse pressure and left atrial size but an association between AIx and left atrial size [21]. Another study reported a significant correlation between cfPWV and left atrial diameter [39]. Brachial-ankle PWV and cfPWV correlated with left atrial diameter in patients with hypertension, end-stage renal disease, and heart failure [53]. One potential explanation for the differences between studies is the differences in the proportion of other factors that influence left atrial size in each of the study population. Left atrial size is larger in patients with hypertension and in the elderly as well as patients with reduced left ventricular systolic function [11, 22]. The proportion of individuals with each of these characteristics likely influenced the relationship between left atrial size and arterial stiffness. Our study consisted of individuals with normal LV systolic function. 

Augmentation index was not associated with diastolic dysfunction in our study. This finding is consistent with two studies [21, 54] but contrasts with the findings of two another studies [55, 56]. While AIx is an indicator of arterial stiffness, pulse wave velocity more closely correlates with adverse cardiovascular outcomes [57]. We did not find any relationship between pulse pressure and diastolic function. Our findings are consistent with some previous data that say that PWV is superior to central and brachial pulse pressures for the detection of diastolic dysfunction in older adults with “preserved” LV ejection fraction [58]. 

 Several possible limitations of this study should be considered. The patient population is relatively small. However, significant correlations were still found. Secondly, the changes in *E/A* ratio with the progression of diastolic dysfunction are such that the initial impairment of diastolic function is manifested as a decreased *E/A* ratio. In long standing disease, changes in ventricular and atrial structure occur, and the diastolic dysfunction may predominantly have a restrictive pattern, which is manifested as an increased *E/A* ratio [6]. Others however disagree with this postulated pattern of progression [8]. The use of decreasing *E/A* ratio as an indicator of diastolic dysfunction is appropriate in this study that consists of a study population without severe cardiac disease. Third, we focused mainly on *E/A* ratio as an indicator of diastolic dysfunction. Mitral valve velocities and myocardial tissue Doppler measurements can provide additional important data on the state of diastolic function. However, each of these measurements has unique limitations in their correlation with more precise measurements of diastolic function [8]. Importantly, *E/A* ratio remains a standard among the field of the detection of diastolic dysfunction. Fourth, subjects were on antihypertensive drugs that can impact on arterial stiffness and minimize the wave reflection thereby altering the relative strength of the relationship between different indices of arterial stiffness and diastolic function [59]. Fifth, we use the term arterial stiffness recognizing that this term may encompass abnormalities in separate measures of arterial compliance, distensibility, pressure augmentation, or wave reflection in different segments of the vasculature.

 In summary, the present study shows that increased baPWV is associated with diastolic dysfunction, measured by *E/A* ratio. Age, SBP, and PP were major determinants of increased baPWV, but the relationship between baPWV and *E/A* ratio was independent of these clinical variables. Our findings show that baPWV is more closely correlated with *E/A* ratio than with other indices of arterial stiffness, namely cfPWV, AIx, ABI, or pulse pressure. The more extensive arterial pathway measured by baPWV in comparison to cfPWV may explain this relationship. These data suggest that measuring baPWV has the utility of infering or suspecting the presence of left ventricular diastolic dysfunction.

## Figures and Tables

**Table 1:** Clinical characteristics of the study population.

Number of subjects	41
Age (years)	68 ± 11
Gender (% men)	66
BMI (kg/m^2^)	26.7 ± 3.6
BSA (m^2^)	1.84 ± 0.22

Hypertension (%)	73.2
Dyslipidemia (%)	48.8
Diabetes (%)	12.2
CAD (%)	22.0

Medications	
*β*-blockers (%)	34.1
ACEIs (%)	36.6
ARBs (%)	7.3
CCBs (%)	24.4
Statins (%)	43.9
Aspirin (%)	53.7
Antiplatelets (%)	14.6
Diuretics (%)	19.5

Heart rate (bpm)	67.3 ± 12.8
SBP (mm Hg)	138.5 ± 18.1
DBP (mm Hg)	78.8 ± 8.4
PP (mm Hg)	59.7 ± 14.2

baPWV (cm/s)	1814 ± 415
cfPWV (cm/s)	1610 ± 500
AIx (%)	20.0 ± 18.3
ABI	1.14 ± 0.12

LAD (mm)	36.8 ± 6.5
LAVI (ml/m^2^)	33.5 ± 11.7
*E* (cm/s)	79.6 ± 26.0
*A* (cm/s)	76.0 ± 25.5
*E/A *	1.15 ± 0.54
	Age < 60: 1.3 ± 0.4
	Age ≥ 60: 1.1 ± 0.6
LVEF (%)	63.3 ± 5.0
LVM (g)	151.2 ± 36.6
LVMI (g/m^2^)	80.3 ± 21.0
LVDD (mm)	45.0 ± 5.1
LVSD (mm)	28.4 ± 5.5
IVST (mm)	10.0 ± 1.4
PWT (mm)	9.7 ± 1.2

BMI: body mass index, BSA: body surface index, CAD: coronary artery disease, ACEIs: angiotensin-converting enzyme inhibitors, ARBs: angiotensin II receptor antagonists, CCBs: calcium channel blockers, SBP: systolic blood pressure, DBP: diastolic blood pressure, PP: pulse pressure, ABI: ankle-brachial index, Aix: augmentation index, cfPWV: carotid-femoral pulse wave velocity, baPWV: brachial-ankle pulse wave velocity, LAD: left atrial diameter, LAVI: left atrial volume index, *E*: peak early diastolic filling velocity, *A*: peak late diastolic filling velocity, *E/A*: *E/A* ratio; LVEF: left ventricular ejection fraction, LVM: left ventricular mass, LVMI: left ventricular mass index, LVDD: left ventricular end-diastolic diameter, LVSD: left ventricular end-systolic diameter, IVS: interventricular septal end-diastolic thickness, and PWT: posterior wall end-diastolic thickness.

**Table 2:** Univariate correlates between echocardiographic variables and indices of arterial stiffness.

	*E/A*		LAd		LAVI	
	*r*	*P*	*r*	*P*	*r*	*P*
baPWV	−0.311*	0.04	0101	0.52	0.008	0.96
cfPWV	−0.040	0.11	0.119	0.45	0.213	0.18
PP	−0.152	0.34	0.260	0.10	0.238	0.13
ABI	0.156	0.32	−0.096	0.55	0.135	0.40
AIx	−0.044	0.78	0.048	0.76	−0.098	0.54
Age	−0.180	0.26	0.313*	0.04	0.210	0.18
SBP	−0.170	0.28	0.144	0.36	0.108	0.50

*r*: pearson correlation coefficient; other abbreviations as in Table 1.

*Indicates *P* < 0.05.

**Table 3:** Multivariate analysis for baPWV.

Model	Unstandardized		Standardized		Sig	95% Confidence	
	beta	S.E.	Beta	*t*		for beta	
*E/A *	−455.5	215.065	−.588	−2.118	.042	−892.604	−18.473
Age	26.9	4.959	.751	5.418	.000	16.791	36.947
SBP	21.5	6.315	.937	3.401	.002	8.644	34.310
PP	−23.7	8.286	−.811	−2.854	.007	−40.489	−6.810

**Table 4:** Multivariate analysis for cfPWV.

Model	Unstandardized		Standardized		Sig	95% Confidence	
	Beta	S.E.	Beta	*t*		for beta	
*E/A *	−21.990	278.229	−.024	−.079	.937	−587.418	543.438
Age	26.356	6.415	.611	4.108	.000	13.319	39.393
SBP	22.997	8.170	.833	2.815	.008	6.395	39.600
PP	−18.876	10.720	−.537	−1.761	.087	−40.661	2.909

SBP is systolic blood pressure.

PP is pulse pressure.
